# Insights from Space: Potential Role of Diet in the Spatial Organization of Chromosomes

**DOI:** 10.3390/nu6125724

**Published:** 2014-12-10

**Authors:** Justin M. O’Sullivan, Malina D. Doynova, Jisha Antony, Florian Pichlmuller, Julia A. Horsfield

**Affiliations:** 1 The Liggins Institute, The University of Auckland, Private Bag 92019 AMC, Auckland 1142, New Zealand; E-Mails: m.doynova@auckland.ac.nz (M.D.D.); f.pichlmuller@auckland.ac.nz (F.P.); 2 Department of Pathology, Dunedin School of Medicine, The University of Otago, P.O. Box 913, Dunedin 9054, New Zealand; E-Mails: jisha.antony@otago.ac.nz (J.A.); julia.horsfield@otago.ac.nz (J.A.H.)

**Keywords:** HiC, proximity ligation, genome organization, nutrition, epigenetics, estrogen, cohesion, CTCF

## Abstract

We can now sequence and identify genome wide epigenetic patterns and perform a variety of “genomic experiments” within relatively short periods of time—ranging from days to weeks. Yet, despite these technological advances, we have a poor understanding of the inter-relationships between epigenetics, genome structure-function, and nutrition. Perhaps this limitation lies, in part, in our propensity to study epigenetics in terms of the linear arrangement of elements and genes. Here we propose that a more complete understanding of how nutrition impacts on epigenetics and cellular development resides within the inter-relationships between DNA and histone modification patterns and genome function, in the context of spatial organization of chromatin and the epigenome.

## 1. Introduction

Studies on the effects of dietary factors on epigenetics have been largely restricted to individual genes, or small groups of genes (Table 1). Despite the limited insight into mechanism offered by these approaches, considerable evidence suggests that nutrition quality and quantity can influence cellular development, growth, the onset of non-communicable diseases, and the health of future generations. The mechanisms by which nutrition impacts on these processes are only now being determined. DNA methylation, histone modifications, and non-coding RNAs seem to play crucial and often central roles in biological pathways that underpin cellular responses to nutrition [1,2,3,4]. Having said that, it is important to be aware that these modifying factors are not determinative by themselves, but rather, contribute to a genomic environment within which other molecules (e.g*.*, transcription factors, DNA binding proteins, and polymerases) cooperate to control the reading, repair and replication of the genome [5]. This genomic environment forms and operates in three-dimensional space.

**Table 1:** An overview of effects of dietary factors on the epigenetic modifications of selected genes. Modified from [6].

Gene Name	DNA Methylation		Histone Deacetylation		Histone Methylation	miRNAs Interference	Dietary Factor	Reference
Agouti	√						folic acid, vitamin B_12_, betaine, choline & genistein, bisphenol A	[7,8]
Axin-Fused	√						folic acid, vitamin B_12_, betaine, choline	[9]
LPLRAP1						√	rice diet	[10]
GLUT4			√				calorie restriction & protein restriction during gestation	[11,12]
P21			√				sulforaphane	[13]
BAX			√				sulforaphane	[13]
IGF2	√		√		√		prenatal exposure to famine & protein restriction during gestation	[14,15]
INSIGF	√						prenatal exposure to famine	[16]
GNASAS	√						prenatal exposure to famine	[16]
MEG3	√						prenatal exposure to famine	[16]
IL10	√						prenatal exposure to famine	[16]
ABCA1	√						prenatal exposure to famine	[16]
LEP	√						prenatal exposure to famine & low calorie diet	[16,17]
POMC			√		√		maternal undernutrition, twinning	[18]
FASN	√						high fat diet	[19]
TNFα	√						n-6 PUFA uptake	[20]
Metastable epialleles (BOLA3, LOC654433, EXD3, ZFYVE28, RBM46ZNF678	√						seasonal periconceptual dietary intakes of methionine, choline, betaine, cofactors (folate, vitamins B2, B6, B12, active B12)	[4]

## 2. Genome Organization

In biology, higher order organization is obvious in the various structures that form at all levels from individual molecules, through to complexes, to cells, tissues, organs, organisms, populations, and ecosystems. Although this organization is obvious, its nature is often difficult to readily define in accessible terms. Here we use the term genome organization to describe the three-dimensional arrangement of the cell’s DNA within the nucleus, organelles, or cellular space (Box 1).

The cell’s chromosomes are a series of covalently connected functional and non-functional elements, genic and inter-genic regions that are subjected to a series of covalent (e.g., methylation) and non-covalent (e.g., nucleosome and protein binding) modifications. A linear view of chromosomes is informed by the well-known helical and repeating polymer structure of DNA [21]. This view is enhanced by limitations of print media and software that reinforce our tendency to represent figures in two dimensions within genome browsers (e.g., UCSC Genome Browser) and manuscripts (e.g., [22,23]). Despite this, it is commonly recognized that DNA is folded to fit into cells. So, how does this folding occur and what is its relevance to nutrition and epigenetics?

## 3. How Are Genomes Organized?

At its simplest level, chromosome folding is the product of three inherent properties: (1) chromosomes are long, aperiodic polymers whose ability to bend and twist is definable and measurable [24,25]; (2) chromosomes have volume, therefore no two chromosomal loci can simultaneously occupy exactly the same space; and (3) chromosomes are confined within a space, the diameter of which is shorter than their fully extended linear length. However, ultimately the folding of chromosomes is constrained by the nuclear factors that bind them (e.g., histones) and restrict their flexibility, or that directly anchor the chromosomes to each other, the lamina, or the nuclear membrane.

Box 1 **Genome organization.**

Determining genome structure is dictated by the biological questions being asked. For example, the linear structure of a genome can be determined by various methods including, but not limited to, sequencing and linkage analyses. By contrast, the high resolution definition of the three dimensional organization of a genome can also be interrogated using different microscopic or molecular methods, each of which answers a different and specific question.

Microscopic methods have been widely used to study chromosome structure and modern methods are passing the diffraction limits for previously unheard of resolution (e.g., [26,27]). However, these methods are currently limited in terms of simultaneously enabling the global positioning of epigenetic modifications and the DNA in a manner that allows the identification of the underlying sequence at all sites in the structure.

There are several different molecular methods that can be used to investigate chromosome structure (e.g., DamID [28,29] and proximity ligation). These methods have the advantage that they simultaneously allow the sequence specific identification of DNA structure across the entire genome. Moreover, they can be combined with Chromatin Immunoprecipitation (ChIP) and other molecular methods (e.g., MeDIP) to incorporate direct measurements of the 3D organization of the epigenetic modifications.

**Figure 1:**
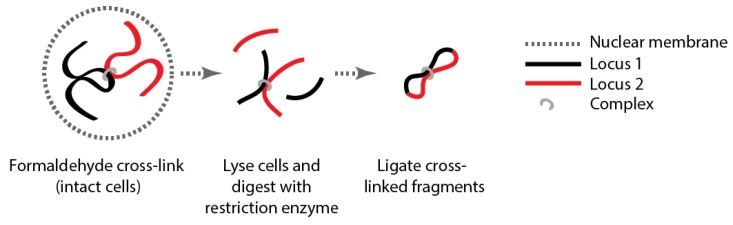
The key steps in proximity ligation.

Proximity ligation (Figure 1) was initially established to investigate gene regulation [30,31]. However, the technique was further developed and popularized by Dekker *et al.* who used it to interrogate the structure of *Saccharomyces cerevisiae* chromosome III [23].

Proximity ligation has been incorporated into a number of different albeit related methodologies (Table 2). These methods generally rely on the same central theme: (1) loci that are held together by some complex (unknown) or are physically contacting each other are chemically tied (cross-linked) together; (2) capture the interacting partners by digestion and ligation; (3) identify the interacting partners using a combination of PCR, microarray, sequencing and bioinformatics (Figure 1). See [32,33,34] for comprehensive reviews on the proximity ligation based methods. Of particular relevance for nutritional studies is the fact that, unless single cell studies are performed [35], proximity ligation only informs on the average genome structure across the cell population in the sample of interest [36].

**Table 2:** The basic suite of proximity ligation methods used for the interrogation of chromosome structure. Modified from [32,33,37].

Method	Key Adaptation	Detects	Reference
3C	*A priori* choice of both interacting sequences	Intereactions between two loci	[23]
4C	*A priori* choice of one interacting sequence	Interactions	[38,39]
5C	Design of primers for indepth interogation of structure of one region	One region	[40]
GCC3C-seq	Sequence all products in ligated library	All interactions in genome	[41,42]
HiC	Enrich for biotin labelled restriction sites after ligation	All interactions in genome	[43,44]
6C ChIA-PET	Enrich for interactions involving protein of interest. Enrich for interactions involving transcription factors	All interactions mediated by protein of interest. All interactions with a given protein	[45,46]
Chip-Loop	Detects the role of specific transcription factors between a known promotor and enhancer	Two regions associated with a given protein	[47]
Enhanced 4C (e4C)	Analysis of transcriptional interactions genomewide	Interactions between transcribing genes	[48]
Associated Chromosome Trap (ACT)	Investigate mechnanisms of transcriptional regulation in *trans*	Detects distant regions interacting or in proximity with specific target	[49]

Current evidence suggests that the inner leaflet of the nuclear membrane, including protein complexes and protein-DNA contacts, is important for the maintenance of chromosome/genome structure. Examples of these components include nuclear pore complexes [28], positioning of telomeres (reviewed in [50]), and lamins [51,52,53]. The inner leaflet of the nuclear membrane is a dynamic two dimensional surface, within which the anchor points (e.g., nuclear pores) move. Movement of these contacts in space can, in turn, lead to positional variability within the three dimensional genome organization; even when cells are in identical environmental conditions. By contrast, DNA interactions with the nuclear lamina, a relatively immobile mesh-like network structure, are thought to contribute to the stability of the metazoan nucleus and contribute to a reduction in variability [51,52,53]. Lamina-interacting chromatin regions are enriched for CCCTC-binding factor (CTCF), consistent with a role for this 11-zinc finger protein as a chromatin boundary determinant due to its insulator properties [53,54].

Chromosomes fold up to form individual territories and show an evolutionarily conserved preference for the formation of intrachromosomal contacts (reviewed in [55]). The formation of these territories is thought to result from the polymer-like nature of the chromosomes and the fact that equilibration of chromosome intermingling takes too long to occur and resolve as cells progress through the cell cycle [56]. However, chromosome territories are not exclusive [57] and the formation of the limited interchromosomal contacts that do occur contributes to the 3-dimensional organization of the genome and the control of nuclear functions [58].

## 4. Is Genome Organization Necessary for Cellular and Subsequent Levels of Function?

Gene regulation by distal regulatory elements (e.g., locus control regions (LCRs)) is thought to be facilitated by direct physical interactions between the LCRs and their gene targets, which help recruit the factors necessary for transcription. These interactions involve looping out of the DNA sequence that intervenes between the LCR and its target [48,59,60]. If the regulatory elements that interact are physically linked on a single chromosome, then alterations to gene regulation can correlate with the formation or breakage of intra-chromosome interactions; thus affecting the organization of the chromosome territory. For example, physical contact between the unmethylated imprinting control region located upstream of the H19 gene and the two flanking regions surrounding the adjacent Igf2 gene (*i.e.*, MAR3 and DMR1) leads to silencing of the Igf2 gene in mouse [61]. Similarly, *cis* looping occurs at the mammalian β-globin locus and this looping is linked to the developmental regulation of the fetal and adult forms of the globin genes [62,63,64]. Forced looping at the globin locus has been shown to be able to over-ride the stringent developmental gene expression program, confirming that looping is a causal event in transcription [65,66]. The ability of forced looping to overcome a developmental program indicates that manipulating loop formation may represent a novel form of therapy [66].

LCRs can also be physically located on a different chromosome from the loci they regulate (e.g., [67]). In these instances, alterations to gene regulation results in the formation or breakage of interactions that occur between chromosomes—in *trans* [68,69]*.* For example, interactions that form between the Interferon-γ (IFN-γ) gene residing on chromosome 10 and part of the *T_H_2* cytokine gene residing on chromosome 11 are an example of *trans* regulation [68]. The formation of this interaction is a characteristic of IFN-γ expressing naïve T helper lymphocytes [68]. How these trans-chromosomal interactions respond to nutritional interventions is poorly characterized.

## 5. Can Chromatin Structure Respond to Nutritional Factors?

Long-range chromatin interactions have been implicated in transcriptional regulation of developmentally and environmentally responsive genes. Some of the best studied examples relate to the developmental regulation of the β-globin locus (reviewed in [62,70]), *T_H_2* [67,68], sonic hedgehog (*Shh*) and *Hox* loci (reviewed in [70]).

The roles of long-range chromatin interactions in environmental responses have been demonstrated for IFN-γ, β-oestradiol and 5 α-dihydrotestosterone and TNF-α inducible genes [71]. In human fibroblast cells it is clear that TNF-α regulated genes that share the same enhancer types are more frequently induced together than expected by chance [71]. Moreover, long-range interactions between these sites are correlated with transcriptional induction [71]. Interestingly, it seems that many of the loops are pre-existing, and cell type dependent [71,72]. The presence of pre-existing loops raises the possibility that the spatial environment set up upon cellular differentiation restricts cell-type-specific responses to specific signal-dependent transcription factors [71]. Despite this, flexibility within cell types allows localized and long-range changes to the chromatin structure to occur.

## 6. Estrogen Exposure and Chromatin Organization

Most nutritional intake is intentional, through food and water. However, some components of our diet are derived inadvertently from environmental pollutants that contaminate crops and ground water. Among these are natural and artificial estrogens, such as Bisphenol A, Diethylstilbestrol, and Genistein, which may be ingested as a result of environmental contamination. Naturally, estrogens are also deliberately consumed as part of hormone-replacement therapy or pregnancy prevention strategies. Inappropriate stimulation of estrogenic signaling pathways can have profound effects on development of the reproductive system, neuronal and gonadal function. Although estrogens have direct gene targets, there is already good evidence demonstrating that exposure to environmental estrogens can result in inappropriate epigenetic modifications (reviewed in [73]. Among the many published examples, Bisphenol A (BPA) inhibits the methylation of imprinted genes during oogenesis [74,75] and affects the expression of microRNA targeting SOX family genes [76], while the endocrine disruptor and pesticide Methoxychlor leads to hypermethylation of *Esr2* and induces *Dnmt3b* expression in the ovary [77]. Studies to date have primarily characterized epigenetic alteration of specific genes and pathways (reviewed in [73]). However, inappropriate activation of estrogen signaling pathways could also have a profound effect on global genome organization, leading to dysregulation of many genes that may not necessarily be estrogen-sensitive.

**Figure 2:**
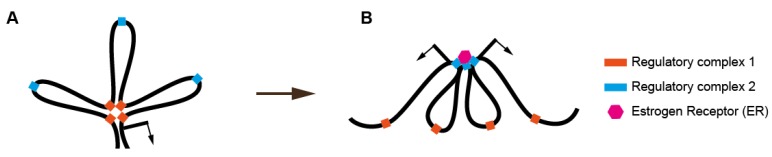
Alterations to the three-dimensional organization of chromatin are linked to transcriptional changes induced by exposure to environmental estrogens. In this illustration, exposure to environmental estrogens is used as an example to demonstrate a potential mechanism by which nutrition could affect 3-D chromatin organization and the transcriptional regulation of multiple genes. (**A**) Transcription from a promoter (arrow) that is regulated in a cell type-specific manner by a regulatory complex assembled by long distance connections that form DNA loops. Regulatory elements outside of the cluster (blue) are not connected and do not drive transcription; (**B**) Inappropriate exposure to estrogen activates the estrogen receptor (ER), which contacts specific regulatory sites (blue) and activates specific gene expression. The formation of an ER-nucleated cluster breaks existing DNA-DNA contacts, and not only inappropriately activates gene expression (arrows) but also disrupts normal gene expression that existed within the original chromatin architecture in A.

Following estrogen stimulation, estrogen receptor alpha (ER) binds genome-wide to estrogen response elements (EREs). Studies in breast cancer cell lines showed that ER frequently binds in conjunction with a protein complex that helps to organize the 3-dimensional structure of the nucleus: Cohesin [78]. Like CTCF, cohesin mediates DNA-DNA contacts and is important for mediating enhancer-promoter communication events that activate gene expression [79]. However, cohesin also maintains DNA contacts within topologically-associated domains (TADs) in the nucleus [80]. This raises the possibility that enhancing ER binding in cells that are not normally exposed to high levels of estrogen might redistribute cohesin to different sites on chromosomes. Redistribution of cohesin would in turn have the capacity to alter global chromatin structure in the nucleus, leading to altered regulation of multiple genes, including those that are not normally estrogen responsive (Figure 2). We have observed that cohesin depletion leads to diverse changes of gene expression in response to estrogen, including a redistribution of DNA-DNA contacts, highlighting the sensitivity of genome structure to chromatin components that modulate transcriptional responses [81].

DNA methylation is also altered by inappropriate exposure to estrogenic compounds (reviewed in [73]), and by itself can lead to altered local (and perhaps global) chromatin structure. For example, exposure to di-ethylhexylphthalate (another by-product from the plastics industry) leads to reduced methylation of maternal imprinted genes *Igf2* and *Peg3* in mouse oocytes [82]. Altered methylation in turn leads to differential binding of CTCF, which means that the normal spatial arrangement of imprinted genes becomes disrupted (see [83]). Therefore, while selected localized epigenetic alterations have been quite well characterized, it is highly likely that more systemic effects on genome organization and function occur as a result of inappropriate exposure to estrogens.

## 7. Organizing the Whole Genome or Part of the Whole?

Metazoan genomes are composed of the nuclear and mitochondrial chromosomes. In terms of relative sizes, nuclear chromosomes are ~3 Mb while the mitochondrial genome is ~16 Kb and it only contains ~37 genes, therefore it is easy to marginalize the contribution that the mitochondrial genome makes to the overall genetic information in the cell. However, given that there can be of the order of hundreds to thousands of mitochondrial chromosomes per cell, the overall contribution the mitochondrial genome makes can be significantly more than is immediately apparent. Interestingly, the ratio of nuclear: Mitochondrial genome copy numbers is linked to development and replication [84,85,86]. Moreover, cell type specific mutations within the mitochondrial genome indicate that selective pressure exists for particular mitochondrial haplotypes [87,88]. How this selective pressure is realized is difficult to imagine when the haplotypes involve mutations that do not occur in known functional elements or coding regions within the mitochondrial genome. However, it is possible that they can be realized through alterations to the genome organization in response to nutritional cues.

Transfer of mitchondrial DNA into the nucleus has occurred over the course of evolution and continues to occur, as evidenced by the presence of recently inserted mitochondrial sequences (*i.e.*, nuclear mitochondrial sequences, NuMTs) within a wide range of eukaryotic genomes [89,90,91]. In Baker’s yeast (*Saccharomyces cerevisiae*) and fission yeast (*Schizosaccharomyces pombe*) it has been shown that mitochondrial DNA forms interchromosomal connections with the nuclear DNA [92,93]. Moreover, these connections are environment, nutrient and cell cycle specific. Finally, the evidence indicates that the connections affect the expression of the nuclear genes—as prevention of connections results in an increase in nuclear gene expression in *S. cerevisiae*, while formation of connections precedes changes in transcript levels in *S. pombe*. Moreover, the quality and quantity of mitochondrial DNA has been shown to affect patterns of nuclear transcription [94,95] and replication [85] in yeast. It remains to be seen whether these connections occur in higher eukaryotes, and if so, whether they have any biological function. However, given the role for mitochondria in nutrient sensing and metabolism, these connections may provide an interesting and novel means of communication between the mitochondria and the nuclear epigenome.

## 8. Conclusions

Responses to environmental cues are driven by changes in the levels, locations, and combinations of transcription factors which act within the three dimensional epigenetic landscape that results from the spatial organization of the genome. Therefore, we contend that a complete understanding how nutrition impacts on epigenetics and cellular development must involve the inter-relationships between DNA and histone modification patterns and genome function in the context of spatial organization of the epigenome.

This hypothesis has clear and significant implications for understanding the mechanisms by which rapid and coordinated functional changes occur within cells in response to environmental factors that affect the epigenome (e.g., the nuclear receptor super-family mediated effects of glucocorticoids, thryroid hormones, vitamins A and D, polyunsaturated fatty acids on nuclear structure and function) [3]. Yet the question remains: How feasible is it to test this hypothesis and its significance in nutritional studies? In our opinion, 4C and ChIA-PET currently represent the most powerful and readily applicable proximity ligation technologies for the study of the effects of known factors (e.g., hormone receptors) in the coordination of nutrient responsive changes in genome structure and function. Ultimately the screening of the nutritional effects on the epigenome of multiple tissues within, or between, individuals is considerably more feasible using a q3C approach. The *a priori* selection of short or long-range chromosome connections for targeting by q3C can be informed by computational interrogations of other datasets (e.g., EnCODE [96]) in combination with information about known risk factors (e.g., single nucleotide poolymorphism identified by genome wide association studies) [97]. Future reductions in sequencing costs and methodological advances will reduce the financial and analytical costs of the application of these techniques to the study of nutritional effects on the epigenome.
